# The cost of monitoring warfarin in patients with chronic atrial fibrillation in primary care in Sweden

**DOI:** 10.1186/1471-2296-8-6

**Published:** 2007-02-26

**Authors:** Ingela Björholt, Stina Andersson, Gunnar H Nilsson, Ingvar Krakau

**Affiliations:** 1 Institute of Clinical Sciences, Göteborg University, Göteborg, Sweden and Nordic Health Economic Research AB, Göteborg, Sweden; 2 AstraZeneca Sverige AB, Södertälje, Sweden; 3 Neurotec Department, Karolinska Institutet, Stockholm, Sweden; 4 Center of Family Medicine, Karolinska Institutet, Huddinge and Department of Medicine, Solna, Sweden

## Abstract

**Background:**

Warfarin is used for the prevention of stroke in chronic atrial fibrillation. The product has a narrow therapeutic index and to obtain treatment success, patients must be maintained within a given therapeutic range (International Normalised Ratio;INR). To ensure a wise allocation of health care resources, scrutiny of costs associated with various treatments is justified. The objective of this study was to estimate the health care cost of INR controls in patients on warfarin treatment with chronic atrial fibrillation in primary care in Sweden.

**Methods:**

Data from various sources were applied in the analysis. Resource consumption was derived from two observational studies based on electronic patient records and two Delphi-panel studies performed in two and three rounds, respectively. Unit costs were taken from official databases and primary health care centres.

**Results:**

The mean cost of one INR control was SEK 550. The mean costs of INR controls during the first three months, the first year and during the second year of treatment were SEK 6,811, SEK 16,244 and SEK 8,904 respectively.

**Conclusion:**

INR controls of patients on warfarin treatment in primary care in Sweden represent a substantial cost to the health care provider and they are particularly costly when undertaken in home care. The cost may however be off-set by the reduced incidence of stroke.

## Background

The prevalence of chronic atrial fibrillation (CAF) has been estimated at 1% of the total population of Sweden [1,2], and a recent study demonstrated a prevalence of 0.6 in primary health care [3]. The corresponding figure from the U.S. for the occurrence of atrial fibrillation has been estimated at 0.9 [4,5]. The prevalence of AF increases as patients grow older. Atrial fibrillation is a potentially disabling and fatal disorder, due to an annual incidence of stroke of 5% [6]. Of all ischemic strokes, about 16% are related to non-valvular AF, and in patients over 75 years of age this frequency increases to one third [6].

Oral anticoagulant drugs, i.e. vitamin K antagonists, are used for the prevention of stroke in CAF and their efficacy has been demonstrated in a large number of studies [6]. However, warfarin has a narrow therapeutic index and to obtain treatment success, the patients must be maintained within a given therapeutic range. This is defined as the International Normalised Ratio (INR) and the most commonly recommended range is INR 2–3. If the INR is below range, the patient will be at risk of thromboembolism and if the INR is above range, the patients will be at risk of experiencing a hemorrhagic event. Furthermore, warfarin interacts with certain types of food, alcohol and many other drugs. Thus the management of patients on warfarin requires frequent monitoring and dose adjustment in order to maximise the time the patient spends within the therapeutic INR range. In Sweden, such controls are usually carried out in dedicated anticoagulation clinics in hospitals or in routine primary care.

In today's climate of increasing scrutiny of health care costs, an analysis of costs associated with the use of warfarin is justified. The cost of warfarin *per se *is low, but INR monitoring is likely to be associated with the use of large resources. Firstly, because INR controls are both frequent and resource-consuming for each patient concerned, and secondly because treatment of warfarin is used in such broad groups of patients. Furthermore, when complications occur, the treatment costs may rise substantially.

The cost-effectiveness has been studied in the prevention of stroke in non-valvular atrial fibrillation, which is the most common diagnosis for warfarin treatment [7-12]. These studies were performed in varying sub-sets of patients, and the conclusions were that treatment with warfarin was cost-effective (and sometimes cost-saving). All studies included an estimate of the cost of warfarin including costs of INR monitoring, but none reported the detailed resource consumption related to INR monitoring. The estimates were therefore either derived from tariffs or gross estimates. All but two were carried out based on data from the U.S. Of the non-U.S. studies, one was from the U.K. and one from Sweden and both were based on management of patients in a hospital setting [10,11].

To be able to estimate the costs of a particular health care activity, various kinds of data from different sources must be available. These include (i) the type and number of health care or other resources that are involved, (ii) the frequency by which such resources are used, (iii) the unit costs of all individual cost items. In the case of the cost of monitoring of warfarin, this is particularly complicated as an INR control is not a defined entity, on the contrary it might involve different kinds of resources depending on different organisations and individual preferences. As many of the patients under warfarin treatment are old and frail, a certain proportion of the patients are subject to domiciliary care.

The objective of this health economic assessment is to estimate the health care cost of monitoring warfarin in patients with CAF managed in primary care in Sweden.

## Methods

The term "INR controls" is below defined as all aspect involved in the monitoring of warfarin, including preparations and follow-up.

### Frequency of INR controls

The frequency of INR controls during the initiation phase and during established treatment was derived from two retrospective studies of electronic patient records [3,13,14]. Both studies were performed in Stockholm, where patients on warfarin routinely are managed in primary care.

One study was performed on patients with CAF during the initiation phase of warfarin treatment (the first three months of treatment) [13]. Twelve primary health care (PHC) centres from five different health care districts in Stockholm County with a registered population of 203, 407 individuals included 144 patients.

In the other study, five PHC centres with a registered population of 75,146 participated. Twenty-five patients with CAF, who had received a minimum of 30 days treatment were randomly selected for a detailed review of the clinical management of warfarin treatment [3,14]. Of those, five patients had been treated for less than three months, one had been on treatment for 11 months and 19 had been on treatment for more than one year. All patients in the initiation phase of treatment were excluded and data from the remaining patients (n = 20) were used to represent the frequency of INR controls in established patients, defined as treatment for 12 months or more. Information on the frequency of INR controls between four and 11 months treatment was not available and an assumption of a linear decline was applied, based on available data on the frequency of INR controls during month three and month 12.

### Resources consumed per INR control

The type and number of resources consumed at INR controls were investigated in two Delphi-panel studies [15]). In such panels standardised techniques are used to systematically collect and collate informed judgments from a group of experts on specific questions or issues [16-20]. The respondents are anonymous to each other and the study is performed in different rounds.

The respondents in both Delphi-panels were health care personnel. One was performed in three rounds and had the objective to study the resource use of patients whose INR controls were carried out onsite at a PHC centre. Thirty-four general practitioners (GPs) and 10 registered nurses (RNs) from 34 PHC centres in Stockholm participated. The other Delphi panel investigated the resource consumption for patients managed in home care. It was performed in two rounds and 49 district nurses (DNs) from all over Sweden were enrolled.

In the application of the Delphi technique in the studies referred to in this article, the expert panel members were randomly selected from GPs and RNs in primary care in Stockholm and DNs in Sweden respectively. The panel members were asked to estimate patient-related time defined as time spent in preparation before the INR control, direct time spent together with the patient and follow-up in connection with the control. Total patient-related time spent by GPs and RNs respectively was regarded as a reflection of resources used in INR controls, apart from the work undertaken by laboratory staff and the cost of transportation to and from patients in home care, which were calculated separately. The costs were estimated by multiplying the various resources by their respective unit cost.

### Unit cost calculation

Unit costs were based on data from 2003, estimated from the perspective of the health care provider and expressed in SEK (SEK 1 = € 0.11). Where costs have been reported in $ or ₤ in publications referenced in this article, the following exchange rates have been used; $1 = SEK 7.61 and ₤1 = SEK13.88. All exchange rates are as of June 30, 2004.

#### Cost of a patient-related hour

Gross payroll expenses were defined as the average annual costs (salary, security fees and pensions) of GPs, RNs and DNs in the County Council of Stockholm in Sweden. It was SEK 868, 977 for a GP, SEK 382,508 for an RN and SEK 412, 482 for a DN.

The average annual working hours in 2003 for full-time employees in the health care sector was 1,576, which was derived from a labour force survey performed by Statistics Sweden (unpublished data). The patient-related time was estimated to be 73% for the GPs. This figure was based on a survey performed by The Swedish Institute for Health Economics in 1999–2000 in which four different specialities were represented including GPs (n = 81) [21]. GPs reported they spend 50% of their total working-hours in face-to-face consultations with patients, and another 23% of their time for administrative work directly related to patients. This means 73% of the working-hours are allocated to patient-related work. We estimated the patient-related time for RNs and DNs to 85% based on the assumption that patient-related time is greater for nurses than for GPs.

Hence the average annual working hours of patient-related time (Ann_pat_TIME) was estimated at 1,150 (0.73*1576) for GPs and 1,340 (0.85*1576) for RNs and DNs.

The gross payroll expenses of a patient-related hour (Gross_payroll_exp_pat_hour) was

Gross_payroll_expensesAnn_pat_TIME
 MathType@MTEF@5@5@+=feaafiart1ev1aaatCvAUfKttLearuWrP9MDH5MBPbIqV92AaeXatLxBI9gBaebbnrfifHhDYfgasaacH8akY=wiFfYdH8Gipec8Eeeu0xXdbba9frFj0=OqFfea0dXdd9vqai=hGuQ8kuc9pgc9s8qqaq=dirpe0xb9q8qiLsFr0=vr0=vr0dc8meaabaqaciaacaGaaeqabaqabeGadaaakeaadaWcaaqaaiabbEeahjabbkhaYjabb+gaVjabbohaZjabbohaZjabc+faFjabbchaWjabbggaHjabbMha5jabbkhaYjabb+gaVjabbYgaSjabbYgaSjabc+faFjabbwgaLjabbIha4jabbchaWjabbwgaLjabb6gaUjabbohaZjabbwgaLjabbohaZbqaaiabbgeabjabb6gaUjabb6gaUjabc+faFjabbchaWjabbggaHjabbsha0jabc+faFjabbsfaujabbMeajjabb2eanjabbweafbaaaaa@59C0@

Hence the "Gross_payroll_exp_pat_hour" was SEK 755 (868,977/1,150) for GPs, SEK 286 (382,508/1,340) for RNs and SEK 308 (412,482/1,340) for DNs.

The balance-sheets of PHC centres include gross payroll costs as well as overhead and administrative costs such as rental and maintenance of the localities, capital costs and telephone. These costs were denoted "other costs" and were added to the payroll expenses to obtain a total cost of a patient-related hour. The accounts of three PHC centres, one big (Gustavsberg), one middle-sized (Österåker) and one small centre (Nyby), showed the average of "other costs" was 40% of total cost.

The following formula was applied for calculation of the total cost of a patient-related hour including "Gross_payroll_exp_pat_hour " as well as "Other costs":

Gross_payroll_exp⁡_pat_hour1−Proportion_other_costs
 MathType@MTEF@5@5@+=feaafiart1ev1aaatCvAUfKttLearuWrP9MDH5MBPbIqV92AaeXatLxBI9gBaebbnrfifHhDYfgasaacH8akY=wiFfYdH8Gipec8Eeeu0xXdbba9frFj0=OqFfea0dXdd9vqai=hGuQ8kuc9pgc9s8qqaq=dirpe0xb9q8qiLsFr0=vr0=vr0dc8meaabaqaciaacaGaaeqabaqabeGadaaakeaadaWcaaqaaiabbEeahjabbkhaYjabb+gaVjabbohaZjabbohaZjabc+faFjabbchaWjabbggaHjabbMha5jabbkhaYjabb+gaVjabbYgaSjabbYgaSjabc+faFjGbcwgaLjabcIha4jabcchaWjabc+faFjabbchaWjabbggaHjabbsha0jabc+faFjabbIgaOjabb+gaVjabbwha1jabbkhaYbqaaiabigdaXiabgkHiTiabbcfaqjabbkhaYjabb+gaVjabbchaWjabb+gaVjabbkhaYjabbsha0jabbMgaPjabb+gaVjabb6gaUjabc+faFjabb+gaVjabbsha0jabbIgaOjabbwgaLjabbkhaYjabc+faFjabbogaJjabb+gaVjabbohaZjabbsha0jabbohaZbaaaaa@703E@

Thus the cost of a patient-related hour based on "Other costs" being 40% was SEK 1259 (755/0.40) for a GP ('GP_hour_COST'), SEK 476 (286/0.40) for an RN ('RN_hour_COST') and SEK 513 (308/0.40) for a DN (DN_hour_COST).

#### Sample and transportation costs

The work undertaken by laboratory staff was not investigated in the Delphi panels. Therefore, tariff prices for taking the sample (SEK 80) and the analysis of the sample (SEK 42) were used in the calculations. These costs were derived from Nyby PHC centre.

The cost for car transportation was estimated to be SEK 2.50 per kilometre for a small or mid-sized car (Swedish Consumer Agency in Stockholm).

### Estimate of costs

#### Cost of INR controls at primary care centres

PHC centres were divided into such centres where GPs were routinely assisted by an RN in the management of INR controls for warfarin patients and such centres where GPs did not co-operate with an RN on a regular basis for these controls. All costs are expressed as mean costs.

The cost of an INR control for GPs with an RN ('GPRN_control_ COST') was

['GP_hour_COST' * ('GPRN_TIME' + 'GPRN_exTIME') +'RN_hour_COST' * ('RN_TIME' +'RN_exTIME')] + 'Sample_COST' + 'Analysis_COST'

where

'GP_hour_COST' and 'RN_hour_COST' have been defined above,

'GPRN_TIME' = Mean time GPs, routinely assisted by a nurse, spend on one INR control,

'GPRN_exTIME' = Mean extra time for GPs, routinely assisted by an RN, when a patient does not appear for a scheduled visit.

'RN_TIME' = Mean time nurses spend on one INR control,

'RN_exTIME' = Mean time for an RN when a patient does not appear for a scheduled visit.

'Sample_COST' = Cost of taking the blood sample by laboratory staff and

'Analysis_COST' = Cost of analysing the blood sample.

Similarly, the cost of an INR control when the GP was not routinely assisted by an RN (GP_control_COST) was

* ['GP_hour_COST'* ('GP_TIME' + 'GP_exTIME')] + 'Sample_COST' + 'Analysis_COST'

where

'GP_hour_COST', 'Sample_COST' and 'Analysis_COST' have been defined before and

'GP_TIME' = Mean time GPs, not routinely assisted by a nurse, spend on one INR control,

'GP_exTIME' = Mean extra time for GPs, not routinely assisted by an RN, when a patient does not appear for a scheduled INR control.

Accordingly the total cost of an INR control when the patient is managed onsite at a PHC centre ('PHC_control_COST) was

('GPRN_PROP' * 'GPRN_control_COST') + (GP_PROP * GP_control_COST)

where

'GPRN_control_COST' and 'GP_control_COST' have been defined before and

'GPRN_PROP' = Share of PHC centres where doctors are routinely assisted by a nurse,

'GP_PROP = Share of GPs, not routinely assisted by a nurse,

#### Cost of INR controls in home care

The total cost per INR home care visit ('HOME_control_COST') was:

['DN_hour_COST' * ('DN_TIME'+ 'DN_exTIME')] + (Transp_COST * Transp_KM) + 'Analysis_COST'

where

'DN_hour_COST' and 'Analysis_COST' have been previously defined and where

'DN_TIME' = Mean time district nurses spend on one INR control visit,

'Transp_COST' = Cost per kilometre by car (Swedish Consumer Agency),

'Transp_KM' = Mean distance per INR control, return trip and

'DN_exTIME' = Extra time per INR control due to INR control visits undertaken in vain.

#### Costs of INR control in primary care

Total cost of an INR control undertaken in primary care ('TOT_control_COST') was

('PHC_PROP' * 'PHC_control_COST') + ('HOME_PROP' * 'HOME_control_COST')

where

'PHC_control_COST' and 'HOME_control_COST' have been previously defined and

'PHC_PROP' = Proportion of INR controls undertaken at a PHC centre and

'HOME_PROP' = Proportion of INR controls undertaken in the patient's home.

#### Cost per patient

Total cost of INR controls during the initiation phase (first three months of treatment), during the first year (including the initiation phase) and during subsequent years of treatment was obtained by multiplying TOT_control_COST with the monitoring frequencies observed in or derived from the two retrospective studies on electronic patient records mentioned above [4,13,14].

### Sensitivity analyses

Although this analysis provides the most accurate assessment of the economic impact of INR controls in primary care available to date, a number of uncertainties in the estimates of resource use and unit costs were identified and explored in a series of sensitivity analyses. The following analyses were performed: (i) reduction/increase of the time GPs (GPRN_TIME and GP_TIME and DNs (DN_TIME) used for INR controls, (ii) the proportion of home visits ('HOME_PROP') was lower/higher, (iii) 'Other costs' were lower/higher, (iv) the costs incurred by laboratory staff ('Sample_COST') and analysis of the sample (Analysis_COST') were lower/higher.

## Results

### Frequency of INR controls

In the study on new patients on warfarin, 1,721 INR controls were registered during a follow-up time of 12,688 days, resulting in an average number of INR controls during the first three months of treatment of 12.38 (95% CI: 11.3–12.6) per patient [13]. The mean number of visits during month three was 2.6.

The group of 20 established patients, from Nilsson et al had a mean follow-up time of 0.55 years in the study [3] and the annual frequency of visits was 16.18 (95% CI:11.37–21.02, resulting in a mean number of 1.35 controls per month.

The number of INR controls between month three and month 12 was estimated at 17.14.

### Resource use per INR controls

In 29% of the PHC centres, an RN was routinely involved in the management of warfarin patients ('GPRN_PROP'). The mean time per INR control was 0.17 hours (10.1 minutes, 95% confidence interval, CI, 5.4; 14.8) for GPs ('GPRN_TIME') and 0.36 hours (21.4 minutes, 95% CI 11.0; 31.8) for the RNs ('RN_TIME'). The mean extra time used for a missed appointment by a patient was 6.6 minutes (95% CI 0.3 – 12.9) for GPs and 4.9 minutes (95% CI 2.0 – 7.8) for RNs. When taking the frequency of missed visits into consideration, 11% for the GPs and 17% for the RNs, the mean extra time for "no-shows" was 0.012 hours/visit for the GPs ('GPRN_exTIME') and 0.014 hours/visit for the RNs ('RN_exTIME'), when allocated to such INR controls which actually took place.

Where the GP managed INR controls without routinely cooperating with an RN, the mean time used for a standard INR control was 0.29 hours (17.6 minutes, 95% CI 10.6;24.6) ('GP_TIME'). The extra time spent due the patient's failure to show up for the scheduled visit was 5 minutes (95% CI 2.7 – 7.3). It was reported to happen in 11% of the scheduled visits and, accordingly, the mean extra time per INR control was 0.009 hours ('GP_exTIME'). In addition to the work undertaken by the GP, and RN where applicable, laboratory personnel was involved in taking ('Sample_COST') and analysing the blood sample ('Analysis_COST').

In the PHC centre Delphi study, the respondents reported that 11.6% of their patients were unable to come to the PHC centre for monitoring of INR. In these cases, the monitoring of the patients was undertaken by DNs in home care.

The study in domiciliary care demonstrated that the mean time spent on INR controls in the patient's home was 1.47 hours (88.2 minutes, CI 95% 76.8 – 99.6 minutes) ('DN_TIME'). On average, the district nurse travelled 13.7 kilometres (95% CI 9.0;18.4) by car for each home visit ('Transp_KM'). In 8.2% of the times, the visit was in vain as the patient was not at home. Each such visit took on average 45 minutes (95% CI 33.1;56.1), representing a mean of 0.059 hours per INR control ('DN_exTIME).

Of all INR controls in primary care, 88.4% were carried out at a PHC centre ('PHC_PROP') and 11.6% in a patient's home ('HOME_PROP').

### Cost of monitoring warfarin

All variables are defined and referenced above.

#### Cost of INR controls at PHC

The cost of an INR control for GPs routinely assisted by a nurse ('GPRN_control_ COST') was SEK 525 ([1,259 * (0.168 +0.012) + 476 (0.357+0.014)] + 80 + 42)

The cost of an INR control for GPs working without routinely being assisted by an RN ('GP_control_COST') was: SEK 503 ([1,259 * (0.293 + 0.009)] + 80 + 42)

The cost of an INR control carried out onsite at a PHC centre (PHCcontrolCO) was SEK 509 ((0.29 * 525) + (0.71 * 503))

#### Costs of INR control in home care

The cost of an INR control in home care (HOME_control_COST) was SEK 861 (513* (1.470 + 0.059) + (2.50 * 13.7) + 42)

#### Cost of an INR control in primary care

The mean cost of an INR control in primary care ('TOT_control_COST') was SEK 550 ((0.884 * 509) + (0.116* 861))

#### Total cost per patient

The costs of INR controls per patient based on varying treatment periods are given in Table I.

**Table 1:** Costs of INR controls/patient in primary care in Sweden. Cost/monitoring visit: SEK 550.

	Length of period	No. of INR moni toring visits	Total cost (SEK)
Initiation phase	3 months	12.38	6,811
First treatment year, including initiation phase	12 months	29.52	16,244
Second year	annual	16.18	8,904*

* undiscounted cost

### Sensitivity analysis

The following analyses were performed: (i) the time used for INR controls was reduced/increased by 5 minutes for GPs ('GPRN_TIME' and 'GP_TIME') and 20 minutes for DNs ('DN_TIME'), (ii) the proportion of home visits was 5% lower/20% higher ('HOME_PROP'), (iii) other cost were 30% (lower)/50% (higher) (iv) the cost incurred by laboratory staff ('Sample_COST') and analysis ('Analysis_COST') of the sample was 20% lower/20% higher. The results are given in Table 2.

**Table 2:** Sensitivity analyses.

	Base case			
	Visit	Months 1 – 3	1^st ^yr	2^nd ^yr*)

Base case	550	6,811	16,244	8,904
Shorter time				
Doctors - 5 minutes				
District nurses - 20 minutes	438	5,417	12,920	7,083
Longer time				
Doctors + 5 minutes				
District nurses + 20 minutes	663	8,204	19,567	10,726
Home visits				
5%	527	6,524	15,559	8,529
20%	580	7,176	17,116	9,382
Other costs				
30%	488	6,044	14,415	7,902
50%	637	7,884	18,804	10,308
Laboratory staff and analysis				
Cost 20% lower	528	6,532	15,578	8,540
Cost 20% higher	573	7,090	16,909	9,269

*) Undiscounted costs

## Discussion

We have used results from four studies to estimate the cost of INR controls in patients on warfarin treatment managed in primary care in Sweden. The results demonstrated that substantial health care resources are consumed in the effort to maintain the patients within their recommended therapeutic INR intervals. In the base-case analysis, the cost per monitoring visit was SEK 550 and the cost per patient for the first three months of treatment was SEK 6,811. Furthermore the cost of INR controls for the first year of treatment and the second year of treatment was SEK 16,244 and 8,904 respectively. In sensitivity analyses, the cost per INR control ranged from SEK 438 to 663, where the lowest and highest figures reflected a scenario where the time for the management of the patient had been decreased and increased, respectively. Consequently, the result of the analysis is sensitive to the correctness of the time reported in the Delphi panel studies.

The annual costs for INR controls have in other studies been reported to be between SEK 2,160 and 8,315 [7-11]. The cost per INR control was SEK 120 – 479 [7,10,11]. These studies had varying objectives, none of which were to report on the cost of monitoring warfarin. Such costs were therefore not computed in a detailed fashion, and some of them may have been inadvertently omitted, while we have been able to measure them in our study. In previous studies the cost of monitoring warfarin was a gross estimate applied as one out of many variables in cost-effectiveness analyses, and few details were given on how it was calculated. In some cases the cost represented the tariff price of the actual test [10], while others had also included a gross estimate of the cost of monitoring [7]. In one study the cost of monitoring had been obtain from a telephone survey of a few pharmacies and laboratories [9]. The methods for estimating the costs related to INR controls were thus subject to large variation. Moreover they were derived from different health care settings and based on estimates from a range of different countries. A direct comparison with the results from our study is therefore inappropriate, although we note that the post-initial-phase costs we obtain are similar to the upper bound found in previous studies and that the costs of the initial phase are quite high. It is also unsuitable to directly compare our results with those of the Swedish study by Gustafsson published in 1992, which reports the lowest estimate of monitoring costs (SEK 120/visit, 2,160 annual costs) [10] as it was based on an estimate of the costs in a hospital setting, which may refer to an anticoagulation clinic although not specifically stated.

The annual number of INR controls in patients with AF has previously been reported to be 13.7 [7], 17.3 [11] and 18 [10] and thus they were in the same order of magnitude for established patients as in our study (16.18).

To our knowledge, the data in our study represent the most detailed analysis of the resources consumed and their costs in the monitoring of warfarin patients that is available to date.

Although our approach represents a comprehensive undertaking, the result has its limitations. The frequency of visits was, for example, based on the actual number of visits as registered in patient records. The observed number may be underestimated as INR control may have been carried out elsewhere, which may have been omitted in the patient records. A further limitation of our study is that it did not include societal costs, as costs incurred by patients and or their care providers were not taken into consideration. The same applies to the provision of transportation for patients unable to organise their own travel to the PHC.

Warfarin is used for the prevention of stroke in CAF-patients. In a recent study the life-time direct costs of stroke in Sweden was estimated to SEK 513,800, including hospitalizations, drugs, outpatient visits, nursing home and domestic aid [22]. The annual incidence of stroke in CAF has been estimated to 5% [6], and warfarin has been demonstrated to reduce this risk by approximately 60% [23]. Taken together, this indicates a potential annual cost reduction of SEK 15,000 per patient treated, due to the reduced incidence of stroke. Our study shows that the cost of monitoring patients after the initiation phase of treatment is SEK 8,900 per year. Hence, the cost of such INR controls might be completely offset by the reduction in the cost of stroke even with the higher costs obtained in this study as compared with previous research. This rough calculation puts the cost of INR controls into perspective, but as it disregards other aspects of the treatment, such as costs and health consequences of complications and the timing of events, the result of the calculation should interpreted with caution. A full health economic evaluation of the cost-effectiveness of warfarin is a complex analysis, where all relevant aspects of the treatment should be included.

## Conclusion

INR controls of patients on warfarin treatment in primary care in Sweden represent a substantial cost to the health care provider. The cost exceeds SEK 16,000 during the first year of treatment and it amounts to almost SEK 9,000 per patient during subsequent years. INR controls are particularly costly when undertaken in home care. However, warfarin prevents stroke in CAF, and the cost of monitoring may be off-set by the reduced incidence of this disorder.

## Competing interests

The study was supported by AstraZeneca Sverige AB. SA is a senior health economist at AstraZeneca Sverige AB.

## Authors' contributions

IB performed the health economic analysis. IB, SA, GHN and IK interpreted the results of the analysis and drafted the manuscript. All authors read and approved the final manuscript.

## Pre-publication history

The pre-publication history for this paper can be accessed here:


